# Spontaneous Calcium Oscillations through Differentiation: A Calcium Imaging Analysis of Rat Cochlear Nucleus Neural Stem Cells

**DOI:** 10.3390/cells10102802

**Published:** 2021-10-19

**Authors:** Johannes Voelker, Christine Voelker, Jonas Engert, Nikolas Goemann, Rudolf Hagen, Kristen Rak

**Affiliations:** Plastic, Aesthetic and Reconstructive Head and Neck Surgery and the Comprehensive Hearing Center, Department of Oto-Rhino-Laryngology, University of Wuerzburg Josef-Schneider-Strasse 11, D-97080 Wuerzburg, Germany; voelker.christine@gmail.com (C.V.); Engert_J1@ukw.de (J.E.); niko.goemann@gmx.de (N.G.); hagen_r@ukw.de (R.H.); rak_k@ukw.de (K.R.)

**Keywords:** neurogenesis, neural stem cells, neuronal oscillations, neuronal maturation, auditory pathway, regenerative capacity

## Abstract

Causal therapies for the auditory-pathway and inner-ear diseases are still not yet available for clinical application. Regenerative medicine approaches are discussed and examined as possible therapy options. Neural stem cells could play a role in the regeneration of the auditory pathway. In recent years, neural stem and progenitor cells have been identified in the cochlear nucleus, the second nucleus of the auditory pathway. The current investigation aimed to analyze cell maturation concerning cellular calcium activity. Cochlear nuclei from PND9 CD rats were microscopically dissected and propagated as neurospheres in free-floating cultures in stem-cell medium (Neurobasal, B27, GlutaMAX, EGF, bFGF). After 30 days, the dissociation and plating of these cells took place under withdrawal of the growth factors and the addition of retinoic acid, which induces neural cell differentiation. Calcium imaging analysis with BAPTA-1/Oregon Green was carried out at different times during the differentiation phase. In addition, the influence of different voltage-dependent calcium channels was analyzed through the targeted application of inhibitors of the L-, N-, R- and T-type calcium channels. For this purpose, comparative examinations were performed on CN NSCs, and primary CN neurons. As the cells differentiated, a significant increase in spontaneous neuronal calcium activity was demonstrated. In the differentiation stage, specific frequencies of the spontaneous calcium oscillations were measured in different regions of the individual cells. Initially, the highest frequency of spontaneous calcium oscillations was ascertainable in the maturing somata. Over time, these were overtaken by calcium oscillations in the axons and dendrites. Additionally, in the area of the growth cones, an increasing activity was determined. By inhibiting voltage-dependent calcium channels, their expression and function in the differentiation process were confirmed. A comparable pattern of maturation of these channels was found in CN NSCs and primary CN neurons. The present results show that neural stem cells of the rat cochlear nucleus differentiated not only morphologically but also functionally. Spontaneous calcium activities are of great relevance in terms of neurogenesis and integration into existing neuronal structures. These functional aspects of neurogenesis within the auditory pathway could serve as future targets for the exogenous control of neuronal regeneration.

## 1. Introduction

Sensorineural hearing loss is one of the most common disabilities in humans [1]. However, causal therapies are still at the beginning of any clinical applicability. Regenerative medicine approaches are, therefore, the focus of current hearing research [2]. Since neural stem cells (NSCs) have been identified in certain central nervous system (CNS) regions in recent years, adult neurogenesis has been discussed as a regenerative approach to neuronal pathologies [3,4]. NSCs are capable of self-renewing and differentiating into all cells of the neuroectodermal lineage [5]. Characterized by three cardinal features, NSCs (a) can generate or derive from neuronal tissue, (b) have the potential for unlimited mitotic self-renewal, and (c) can form progenitor cells and differentiate into other cell types by asymmetric division [6]. Neural progenitor cells (NPCs) also have a limited potential for self-renewal and are described in the CNS as line-specific NPCs [5,7]. The functional aspects of neurogenesis, such as endogenous remodeling and regenerative processes, have been critically discussed since their first description [8,9].

The cochlear nucleus (CN) is located at the lateral side of the mammalian brainstem and is the first processing station for auditory information on the central nervous system. The CN consists of different subnuclei: the posteroventral (PVCN), the anteroventral cochlear nucleus (AVCN), and the dorsal cochlear nucleus (DCN). After exiting the cochlea, the auditory nerve enters the brainstem. It bifurcates with one branch synapsing in the posteroventral and dorsal cochlear nucleus and the other innervating the anteroventral CN. [10]. Tonotopically organized fibers reach the CN of the similarly organized spiral ganglion cells, the first auditory neurons, originating from the cochlea [11]. The CN subnuclei each have distinct electrophysiological functions. Parts of the CN provide the frequency coding of the auditory input, time measurements of the signal, and sound localization [12,13]. In the CN, the second auditory neurons arise, transmitting the acoustic information to higher levels in the brainstem, the olivary complex, the nucleus of the lateral lemniscus, and the inferior colliculus.

In addition to degenerative processes in the auditory pathway [14,15], especially in the auditory brainstem nuclei, tumor and nontumor diseases also lead to hearing impairment. The most common tumor-associated damage to the auditory nerve and the structures at the cerebellopontine angle is caused by vestibular schwannoma [16]. Nontumor injuries include ischemic lesions from occlusion of the vertebrobasilar artery [17], damage from viral infections, traumatic lesions—as in temporal bone fractures—damage after meningitis, and congenital malformations [18]. It is known that early postnatal cochlear damage and deafferentation impact the development of the CN and can lead to its atrophy [19]. A degenerative effect on the CN also was shown in animal models in later age groups [20] and was age-associated [21]. A neuroregenerative approach could potentially have a positive impact on auditory rehabilitation. If there is retrocochlear damage to the auditory pathway in the area of the auditory nerve, rehabilitation with a brainstem implant may be possible [22]. With the auditory brainstem implant (ABI), electrodes are placed on the CN and can generate auditory impressions via electrical stimulation of the neurons. The ABI has not yet achieved the same level of benefit as cochlear implants. It can improve directional hearing and lipreading; an open speech understanding is achievable only in rare cases [18,23]. A causal therapy for these cases is not yet available. Neuronal regeneration by induction of neuronal stem cells would be a potential approach to causal treatment. In addition, it is conceivable that the neural interaction with ABI electrodes could be improved by an exogenous influence on neural plasticity by CN NSCs.

NSCs have recently been described in the postnatal cochlear nucleus. The cells displayed cardinal features of NSCs, particularly the capacity to undergo self-renewal by mitosis as well as the differentiation in NPCs and all cells of the neuronal lineage in the rat [24] and mouse [25]. Furthermore, the CN maintains a neurogenic potential until adulthood, shown by the ongoing capacity of neurosphere formation, BrdU incorporation, and detection of progenitor cells by distinct markers [26]. CN NSCs were able to differentiate after transplantation into neuronal tissue forming all neuronal lineage cells [27]. Findings from in vitro and in vivo analyses showed that spontaneous neuronal activity is an essential modulator for processes of structure formation, neuronal regeneration, and neurogenesis [28]. In neural maturation, the synaptogenesis and myelogenesis of sensory cortices are dependent on early neuronal activity [29].

At rest, the intracellular Ca^2+^ concentration is 10^−5^–10^−4^ mmol/L, while the extracellular concentration is about 2 mmol/L. An abrupt increase in intracellular spatial concentration affects several biological processes by changing the conformation of proteins or enzymes [30]. The neurotransmitter transmission of central synapses is calcium-triggered by influencing vesicle exocytosis [31]. The intracellular local calcium distribution influences synaptic activity and strength with voltage-dependent calcium channels [32]. Another functional factor is clusters of voltage-dependent calcium channels and voltage- and calcium-dependent potassium channels, which also play an essential role in the hair cells of the vestibular system [33]. Local contact with calcium channels can have a selective effect on target structures [34]. With the help of signal cascades, the signal can also be relayed outside the specific microdomain and influence gene expression and thus neuronal development [35].

The store-operated calcium channels (SOC) provide an important mechanism for cytoplasmic calcium influx. These membrane ion channels are activated by the elimination of calcium from the endoplasmic reticulum [36]. Such calcium signals influence cell motility and gene expression [37]. The calcium release-activated channels (CRAC) are the best-characterized representatives of the SOCs [38]. They are ubiquitous and play an essential role in calcium-regulated functions of the central nervous system [39,40,41]. In particular, CRAC calcium channels significantly influence neurogenic processes, the proliferation and cell migration of stem and progenitor cells [42].

The calcium ion (Ca^2+^), as a second messenger, also plays a central role in neurogenesis. Spontaneous neuronal activity and the associated intracellular Ca^2+^ signals frequently occur during early perinatal neural development [43,44], and calcium signals play an essential role in neurogenic proliferation [45] and differentiation [46]. These subsequent effects of calcium signals are attributed to regulating the activity of signal proteins and transcription factors. By controlling the cytoskeleton dynamics, the intracellular calcium activity also influences neuronal motility and thus migration during neurogenesis and the formation of axons and dendrites [47]. These spontaneous calcium oscillations are generated by the periodic influx over the cell membrane in interaction with the release from intracellular calcium stores [48]. Voltage-gated calcium channels and neurotransmitter-sensitive receptors, the glutamate and GABA receptors, are essentially involved in these processes [49,50]. Calcium oscillations seem to have an advantage over static signals with these signal cascades due to their superior signal-to-noise ratio [51,52]. In addition, the modulation of the frequency, amplitude, and total duration of the signal enables a sensitive and specific response of the effector proteins [51,53].

The calcium-based signal cascades are already crucial in early neurogenesis. The differentiation of the embryonic neuroectoderm from the ectoderm and thus the formation of neuronal progenitor cells depends on the neuroectoderm-specific gene expression [54] —which can be induced by calcium signals [55]. Neural induction in mammals is essentially dependent on intracellular calcium stores, and thus on the CRAC channel family [45]. One of the key mechanisms is the regulation of the expression of specific genes by calcium signals that code for neurogenesis-related transcription factors—such as the basic helix–loop–helix transcription factors Sox-2 and NeuroD [56,57,58]. These signaling pathways come into play in both embryonic and adult neurogenesis and gliogenesis.

The gene expression interplay occurs through direct interaction with transcription factors or through calcium-sensitive proteins, which interact indirectly. The calcium-triggered influence on the stages of neurogenesis differs within the specific steps. In the early phase of recruitment and proliferation, cholinergic receptor pathways and CRAC channels are fundamental [59]. Voltage-gated calcium channels [60,61] and NMDA receptors [49,57] primarily influence the later stages of neurogenesis: differentiation, migration, and maturation.

In addition to the proliferation and migration of NSCs, neuronal differentiation is one of the most critical steps controlled by calcium signals [62]. The expression of specific ion channels, the associated neuronal excitability [63], and axonal and dendritic growth [63,64,65] are key processes of neurogenesis initiated in this way. These processes are guided by the influence of spontaneous calcium oscillations on the induction of transcription factors, which cause the expression of specific ion channels and neuron-specific receptors [66,67].

A silent neurogenic niche has also been detected in the cochlear nucleus, although its functional development is not yet known. It is unspecified whether spontaneous calcium oscillations occur during the early differentiation of CN NSCs and whether these play a role in maturation. It is also unclear whether intracellular differences in CN NSCs occur, and if they can be influenced exogenously. Therefore, it is unclear whether, in an early phase of neurogenic differentiation in the CN, modulable calcium channels influence the spontaneous calcium oscillation and can therefore be considered a possible target for influencing the behavior and function of NSCs. The current study aimed to examine the differentiation processes of CN NSCs in the early phase of maturation through spontaneous calcium-triggered activity. The basic spontaneous calcium activity of CN NSCs in the course of differentiation was analyzed by live-cell imaging. The function of different calcium channels was investigated in rat CN NSCs in relation to primary CN neurons by specific calcium channel inhibitors. These findings are intended to broaden the understanding of the function and the development of the auditory system and to pave the way for new therapeutic approaches regarding the potential regenerative capacity of the CN

## 2. Materials and Methods

### 2.1. Tissue Preparation, Cell Culture and Neurosphere Assay

All experiments were conducted according to the national guidelines for the care and use of laboratory animals (§8). All experiments described in the manuscript were carried out exclusively as organ removal. Removing organs from the animal after sacrifice is, as per §6 Abs. 1 No. 4 (German Animal Welfare Act), subject to a notification requirement but has not been and cannot be approved as an animal experiment.

The number of sacrificed animals per species per year has to be given to the local authorities. Accordingly, 36 sacrificed Sprague Dawley rats were reported to “Regierung of Unterfranken”.

PND6 CD/Sprague Dawley rats (Charles River^®^, Wilmington, MA, USA) were sacrificed by cervical dislocation, the CN was microscopically dissected and enzymatically dissociated in Accutase (Gibco^®^, Thermo Fisher Scientific^®^, Grand Island, NE, USA). Free-floating NSC cultures were carried out according to the protocols previously described [24,26,68,69]. The dissociation of neural tissue was performed enzymatically in Accutase (Gibco^®^, Thermo fisher scientific^®^) for 30 min at 37 °C in a ThermoMixer^®^ (Eppendorf^®^, Hamburg, Germany). The suspension was triturated every 10 min with a 500 µL pipette. Then, it was centrifuged (1000 rpm, 5 min), and the pellet was suspended in neural stem-cell medium (NSC-medium), containing serum-free Neurobasal^®^ (Thermo Fisher Scientific^®^, Grand Island, NE, USA), 1% GlutaMAX supplement (Invitrogen^®^, Grand Island, NE, USA), B27 supplement without retinoic acid (Invitrogen^®^, Grand Island, NE, USA) and 1% penicillin/streptomycin (Invitrogen^®^, Grand Island, NE, USA). The recombinant murine growth factors EGF (10 mg/mL) (Peprotech^®^, Hamburg, Germany) and bFGF/FGF-2 (10 ng/mL) (PeproTech^®^, Hamburg, Germany) were added. The number of cells in the individual samples was determined using the Neubauer hemocytometer (ZK06, Hartenstein^®,^ Wuerzburg, Germany). Viable cells were determined by staining with trypan blue (0.4%, # 93595, Sigma-Aldrich^®^, St. Louis, MO, USA). Free-floating cell cultures were generated in hydrophobic cell culture flasks (T25, CELLSTAR^®^, filter top, 25 cm^2^, Greiner Bio-One^®^, Monroe, NC, USA) at 37°C and 5% CO_2_. The number of primary spheres was determined after 4 weeks in culture and recalculated for about 1000 cultured cells.

For analyses, neurospheres were carefully aspirated from the free-floating cell cultures with 5 mL autopipettes (accu-jet pro, Brand^®^, Sigma-Aldrich^®^, St. Louis, MO, USA) and plated onto glass coverslips (78.5 mm^2^, Hartenstein, precoated with poly-D-lysine (100 μg/mL, SERVA Electrophoresis^®^, Heidelberg, Germany) and laminin-1 (10 µg/mL, BD Biosciences^®^, Heidelberg, Germany). The spheres were cultivated in 4-well dishes (Greiner Bio-One^®^, Monroe, NC, USA), each with 100 µL of NSC medium per well. The integrity of the plated spheres was checked with an inverted transmitted light microscope (Leica^®^ DMI-8, Wetzlar, Germany). Cultures were then incubated at 37°C/5% CO2 for the intended period. The medium was changed every two days to fresh NSC medium after careful aspiration of the used medium with Pasteur pipettes.

Up to differentiation day 0 (DIF 0), the neurospheres were incubated in NSC medium (Neurobasal, B27, GlutaMAX, EGF, bFGF; (Thermo Fisher Scientific^®^, Grand Island, NE, USA)). For cell differentiation, the individual cells were plated in differentiation (DIF) medium consisting of Neurobasal (Thermo Fisher Scientific^®^), GlutaMAX (Invitrogen^®^), and B27 with retinoic acid (Invitrogen^®^). The 2-D culture was carried out on glass coverslips (ibidi^®^, Graefeling, Germany, gridded glass coverslips grid-500) or 35 mm gridded glass-bottom µ-dishes (ibidi^®^ Graefeling, Germany) coated with laminin-1 (1:100 in 0.05 M D-PBS) and poly-D-lysine (PDL; 1:100 in 0.05 M D-PBS) with a density of 100 cells/mm^2^. A series of 2-D in vitro cultures was carried out to analyze the growth cones (GC), which were particularly suitable for identifying and displaying them. For this reason, a comparatively lower cell density was plated out on the dishes (70 cells/mm^2^) for the investigation of GCs.

For the study of primary CN neurons, the freshly prepared tissue was dissociated with 200 μL Accutase^®^ (Sigma-Aldrich^®^, St. Louis, MO, USA) each for 30 min at 37 °C in a thermomixer. It was then centrifuged at 500 rpm for 5 min, and the supernatant was suctioned off. The single cells were then resuspended in a fresh Neurobasal^®^ (Sigma-Aldrich^®^, St. Louis, MO, USA) medium (Thermo Fisher Scientific^®^, Grand Island, NE, U.S.A) with B27^®^ and GlutaMAX^®^ (Invitrogen^®^, Grand Island, NE, USA). After cell counting, the cells were plated onto glass coverslips coated with laminin-1 and poly-D-lysine for further immunocytological analyses. For calcium imaging experiments, the cells were plated in gridded Petri dishes (ibidi^®^, Graefeling, Germany) and analyzed by the protocols for NSCs.

### 2.2. Calcium Imaging, Loading Protocol, and Immunocytochemistry

Plated cells were loaded with the calcium-sensitive fluorophore Oregon Green BAPTA, AM (OG; O6807; Molecular Probes^®^, Eugene, OR, USA) for calcium imaging analyses. A suspension of 0.5 mL HBSS (without Ca^2+^), 0.5 μL OG solution, and 0.5 μL Pluronic-F-127 solution was used for loading. The cell medium was aspirated from the coverslips and replaced with 250 μL fluorophore solution at 37 °C. Cells were incubated for 15 min and thoroughly rinsed by HBSS solution (with Ca^2+^) several times. Calcium imaging (CaI) was performed using a 40 X immersion objective (Zeiss^®^, Oberkochen, Germany) with a Till Photonics^®^ System and TILLvisION^®^ V4.00 software, T.I.L.L. Photonics, Graefeling, Germany, at an excitation wavelength of 488 nm. The repetition rate was 2400 cycles, with a 118 ms measure-time per cycle. With a high-speed CCD camera, 2400 images were recorded per measurement at an average rate of 8 Hz and 8 ms exposure time. The specific calcium channel inhibitors ω-conotoxin MVIIC (CTX; 2.6 M), nifedipine (Nif; 5 µM), SNX-482 (SNX; 0.2 µM) and kurtoxin (Kur; 0.5 µM) (Biotrend^®^, Cologne, Germany) were used. As a control, the inhibitors were replaced by HBSS (with Ca^2+^). After beginning the CaI measurements, the corresponding inhibitor or HBSS was carefully pipetted in at specific times.

CaI was followed by an immunocytological examination of the analyzed cells after fixation with a 4% paraformaldehyde solution (PFA in 0.1 M PBS). Primary antibodies: mouse monoclonal against Nestin (1:800; #MAB353, Millipore^®^ Burlington, MA, USA), rabbit polyclonal against β-III-tubulin (1:2000; #Ab18207, Abcam^®^ Waltham, MA, USA); secondary antibodies coupled to Alexa Fluor A488 and A555 (1:1000, # A11001, # A11008, Thermo Fisher), polyclonal rabbit against Ca^2+^-channel (α_1_ subunit)-pan (1:400, # C1103, Sigma-Aldrich^®^, St. Louis, MO, USA) and 5 µg/mL DAPI (1:5000, D9542, Sigma-Aldrich^®^, St. Louis, MO, USA)—according to the protocols previously described [26,27,70]. With the help of the numbered grid markings on the coverslips and Petri dishes, the positions of the examined cells and cell clusters were determined. After fixing the cells and immunocytological staining, the exact position of the cells was found with the help of the coordinates. In addition, the photomicrographs from the calcium imaging analyses were morphologically compared with the immunocytological images to precisely identify the cells examined earlier.

### 2.3. Data Analysis and Image Processing

The CaI scans were analyzed by ImageJ V.1.49 h software. The analysis was carried out based on the generated image stacks according to a described, established method [71]. First, the image normalization for the background fluorescence took place over a time window of 20 s (i.e., 300 frames). Thus, relative values of F/F_0_ over time were determined to be stable against local or slow temporal fluctuations. Baseline noise was reduced with a Gaussian filter (5 × 5 filter mask). The signal-to-noise ratio of a peak (event) was defined as: SNR = (F_max_/F_0_ − median F/F_0_)/σ_noise_. Maximum F/F_0_ is the largest value of F/F_0_ during an event, the median F/F_0_ is the average F/F_0_ of event-free baseline noise, σ noise is the standard deviation of the event-free baseline noise. Active cells or compartments were marked as region of interest (ROI) in maximum intensity projections. The calcium transients were quantified in the image sequences within this ROI. All fluorescence curves, which were output by ImageJ using the ROI were viewed individually and double blinded. A threshold value was set at the maximum value minus noise tolerance, and the area around the maximum above the threshold was analyzed. ImageJ then outputs and counts a multipoint selection with one point above each specific maximum. This procedure made it possible to identify irregular intervals or interference signals and exclude false-positive or false-negative values.

Calcium oscillations were analyzed in a synopsis of the values and their peaks with the acquired raw data photos over time and then transferred to spreadsheets. All data were compiled using Microsoft Excel 2021 V16.50 (Microsoft Corporation, Redmond, WA, USA) spreadsheets and statistically analyzed by GraphPad^®^ Prism 8.4.0 software, Graphpad Software, Inc., San Diego, CA, USA). First, a column analysis (D’Agostino–Pearson omnibus normality test) was performed to determine whether a Gaussian normal distribution of the data was present. Subsequently, data were analyzed using the ordinary two-way ANOVA test followed by the Tukey multiple comparison test. A *p*-value < 0.05 was considered to be statistically significant. Reproducible results were obtained from three or more samples. If the data followed a Gaussian normal distribution, mean and standard error of the mean (SEM) are displayed. The final image composition was performed using Adobe^®^ InDesign CC 2021 v16.2.1 software (Adobe Inc., San Jose, CA, USA).

## 3. Results

### 3.1. Differentiating CN NSCs Show Spontaneous Calcium Oscillations

**Figure 1:**
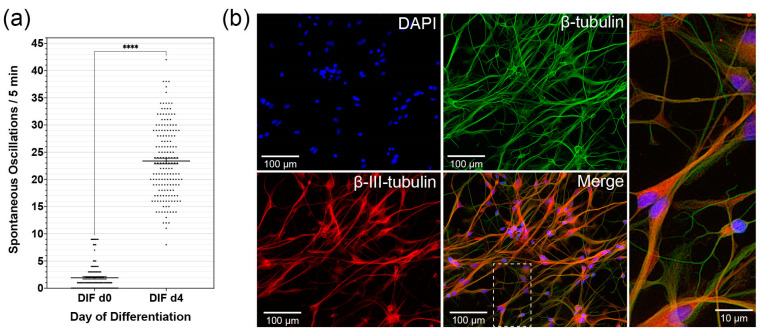
Spontaneous calcium oscillations during CN NSC differentiation. (**a**) Analysis of the spontaneous calcium oscillations by calcium imaging from DIF d0 to d4. (**b**) Representative microphotographs of the immunocytological analysis on DIF d4: DAPI stains the cell nuclei, b-tubulin the cytoskeleton, β-III-tubulin identifies differentiated neurons. Data are shown as mean ± standard error of the mean; asterisks indicate the significance level: **** *p* < 0.0001.

CN NSCs were grown in 2D cultures in DIF medium for specific periods. This was followed by the CaI analysis with Oregon Green^®^ BAPTA-1 (Molecular Probes^®^, Eugene, OR, USA). Over the differentiation period from DIF day 0 to day 4, there was a significant increase in spontaneous calcium oscillations within the measurement periods of 5 min. Calcium peaks were measured for DIF d0: 1.9 ± 0.2 (*n* = 93 cells; mean ± SEM), for DIF-d 4: 23.4. ± 0.5 (*n* = 167 cells; mean ± SEM) (Figure 1a). The number of spontaneous calcium oscillations significantly increased within the differentiation period shown (*p* < 0.0001; δ = 21.48 ± 0.62). The analyzed cells were immunocytologically stained later. The cells previously measured in the CaI were identified with the neuronal marker β-III-tubulin (Figure 1b).

### 3.2. Spontaneous Calcium Activities in the Maturing CN NSC Subregions

**Figure 2:**
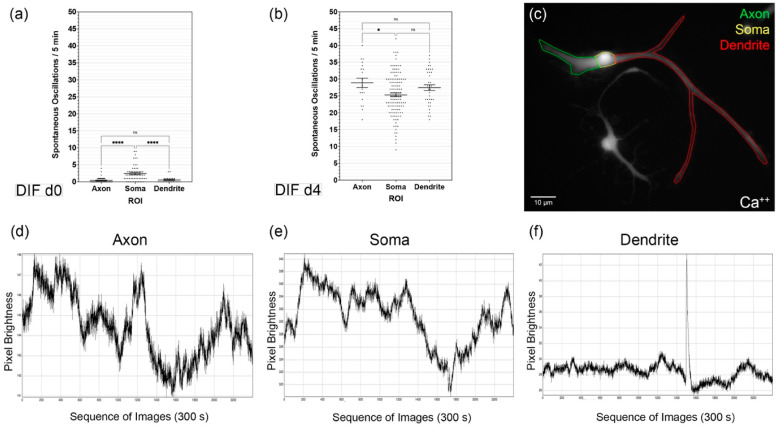
Analysis of spontaneous calcium oscillations in different CN NSC subregions of the cells: (**a**) Spontaneous calcium oscillations in axons, somata, and dendrites from DIF d0 and DIF d4 (**b**) over 5 min. Results are shown as mean ± standard error of the mean. (**c**) Representative photomicrograph in the calcium imaging analysis of NC NSCs with the labeling of different regions of interest (ROI). (**d**–**f**) Representative data of calcium imaging sequences in the corresponding ROI. The individual fluorescence intensity values of Oregon Green^®^/Ca^2+^ as dimensionless values over 300 s/5 min are shown. * *p* < 0.05 and **** *p* < 0.0001, ns = not significant.

During the differentiation phase, measurements of the spontaneous calcium oscillations in the different cell regions were made (Figure 2c). For this purpose, image sequences over 300 s were analyzed for each region and cell (Figure 2d–f). The distinction between axonal and dendritic cell processes was based on morphological criteria. Axons were identified as singular cell processes, starting from an axon hill, possibly with a treelike terminal branch. Branchlike cytoplasmic processes were identified as dendrites. It was found that the significantly highest proportion of spontaneous oscillations occurred in the soma at d0 of differentiation: 2.4 ± 0.3/300 s (mean ± SEM; *n* = 60 cells) (*p* < 0.0001; δ_axon/soma_ = −2 ± 0.33; δ_soma/dendrite_ = 1.87 ± 0.41). The axons showed 0.43 ± 0.12 (mean ± SEM; n = 46 cells) and the dendrites 0.57 ± 0.19 (mean ± SEM; *n* = 23 cells) spontaneous oscillations/300 s (Figure 2a). For DIF d4, significant increases in these values were determined in all regions (*p* < 0.000001; δ_axon_ = 28.46 ± 0.9; δ_soma_ = 22.91 ± 0.83; δ_dendrite_ = 26.91 ± 1.13): in the somata 25.34 ± 0.56 oscillations/300 s occurred (mean ± SEM; *n* = 125 cells), in the axons 28.89 ± 1.4 (mean ± SEM; *n* = 19 cells) and in the dendrites 27.47 ± 0.9 (mean ± SEM; *n* = 36 cells) (Figure 2b). The significantly largest number of spontaneous calcium oscillations was measured in the area of the axons. Thus, the number of spontaneous Ca^2+^ oscillations up to DIF d4 increased in the somata by 1056%, in the axons by 6719%, and in the dendrites by 4819%.

### 3.3. Analysis of the Growth Cone Activities

**Figure 3:**
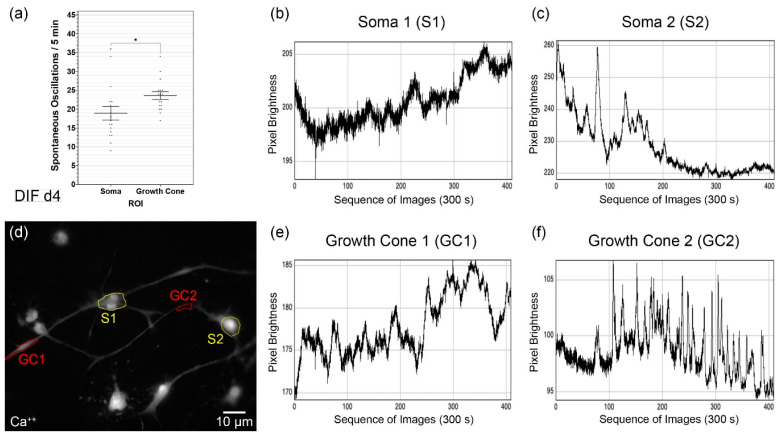
Spontaneous calcium oscillations in the analysis of soma and growth cone of CN NSCs on DIF d4 (**a**). Results are shown as mean ± standard error of the mean; asterisks indicate the significance level: * *p* < 0.05. (**b**,**c**) Representative results of the CaI raw data of somata over 300 s/5 min. (**d**) Representative photomicrograph of CaI fluorescence analysis of associated CN NSCs. (**e**,**f**) CaI raw data of the growth cones over 300 s/5 min.

To investigate the different spontaneous excitations of the soma (Figure 3b,c) and growth cones (Figure 3e,f) of the differentiating neuronal cells, they were analyzed separately (Figure 3d). Image sequences were recorded over 300 s. During the measurement period, a mean of 18.9 ± 1.8 spontaneous Ca^2+^ oscillations were determined in the somata (mean ± SEM; *n* = 17 cells), and the growth cones showed 23.6 ± 1.02 (mean ± SEM; *n* = 17 cells) (Figure 3a). Thus, in the growth cones on DIF d4, spontaneous Ca^2+^ activity was on average 1.25× and significantly higher than in the somata (*p* = 0.032; δ = 4.65 ± 2.1).

### 3.4. Influence of Specific Voltage-Gated Calcium Channel Inhibitors on Primary Neurons and NSCs of the Cochlear Nucleus

**Figure 4:**
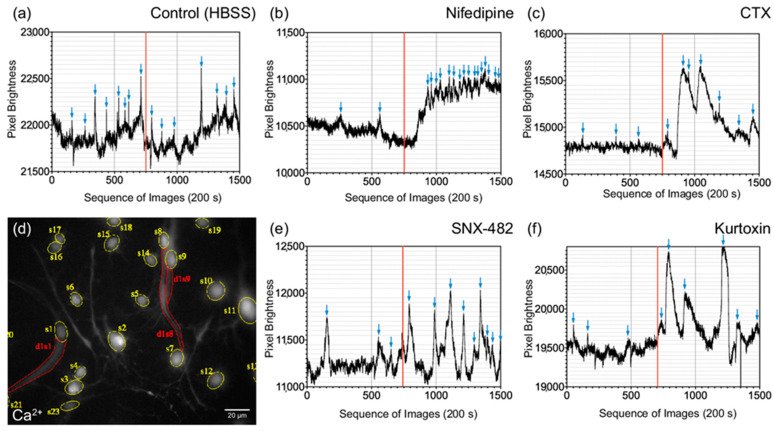
Calcium Imaging Analysis of CN NSC spontaneous oscillations with the application of specific calcium channel modulators: (**a**) control solution (HBSS), (**b**) nifedipine, (**c**) ω-Conotoxin (CTX), (**e**) SNX-482, and (**f**) kurtoxin. The red vertical bar indicates the time of application of the corresponding substances. Blue arrows mark the optically controlled calcium maxima of the spontaneous oscillations. The specific fluorescence intensities over 200 s are shown as dimensionless values. (**d**) representative photomicrograph of CN NSC CaI (Calcium Indicator: Oregon Green BAPTA-1) with tagging of different ROIs.

To analyze the spontaneous calcium activity and the influence of specific calcium channel blockers, dedicated CaI analyses were carried out on DIF d4 (Figure 4d). CN NSCs were rated in relation to primary CN neurons. Ca^2+^ oscillations were measured before application and after application of the substances (Figure 4a–c,e,f).

**Figure 5:**
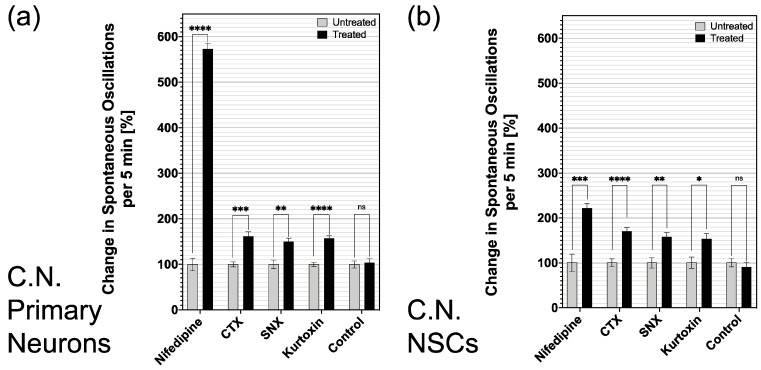
CaI analysis of the influence of specific calcium channel inhibitors on the spontaneous oscillations of primary CN neurons (**a**) and CN NSCs (DIF d4) (**b**). The percentage changes in spontaneous Ca^2+^ oscillations are shown in periods of 5 min—in each case before (untreated) and after application (treated) of the specific substances or the control. Nifedipine, ω-conotoxin (CTX), SNX-48, and kurtoxin were effective in both primary CN Neurons, as well as in CN NSCs (DIF d4), showed a significant increase in Ca^2+^ oscillations. The control solutions (HBSS buffer) did not significantly change the oscillation rate in either analysis. Data are shown as mean ± SEM; asterisks indicate the significance level: * *p* < 0.05, ** *p* < 0.005, *** *p* < 0.001, and **** *p* < 0.0001.

Adding the control solution (HBSS buffer), both primary CN neurons and CN NSCs did not result in a significant change in Ca^2+^ oscillations: 7 ± 0.5 vs. 7.2 ± 0.7/150 s (*n* = 32; mean ± SEM) for primary neurons and 6 ± 0.54 vs. 5.4 ± 0.6/150 s (*n* = 21; mean ± SEM) at CN NSCs. After applying the L-type Ca^2+^ channel inhibitor nifedipine, there was a significant increase in spontaneous oscillations in primary neurons and NSCs: 1.6 ± 0.2 vs. 9.1 ± 1.6/150 s (*n* = 44) at CN neurons (+569%) and 3.4 ± 0.6 vs. 7.4 ± 0.8/150 s (*n* = 14) at CN NSCs (+ 218%). ω-Conotoxin (CTX) also caused a significant increase in Ca^2+^ oscillations in neurons and NSCs: 5.8 ± 0.3 to 9.3 ± 0.9/150 s (*n* = 49) in CN neurons (+160%) and 4.4 ± 0.4 to 7.5 ± 0.6/150 s (*n* = 40) in CN NSCs (+170%). After adding the R-type Ca^2+^ channel blocker SNX-482, a significant increase in Ca^2+^ oscillations from 3.8 ± 0.4 to 5.6 ± 0.5/150 s (*n* = 38) was measured in CN neurons (+147%) and from 4.1 ± 0.5 to 6.5 ± 0.6/150 s (*n* = 35) in CN NSCs (+159%). The T-type Ca^2+^ channel blocker kurtoxin caused a significant increase in spontaneous Ca^2+^ oscillations from 6.4 ± 0.3 to 10 ± 0.6/150 s (*n* = 63) in CN neurons (+ 156%) and from 2.2 ± 0.3 to 3.3 ± 0.4/150 s (*n* = 26) in CN NSCs (+ 150%) (Figure 5a,b).

### 3.5. Differentiated CN NSCs Have a Specific Sensitivity to Calcium Channel Inhibitors

**Figure 6:**
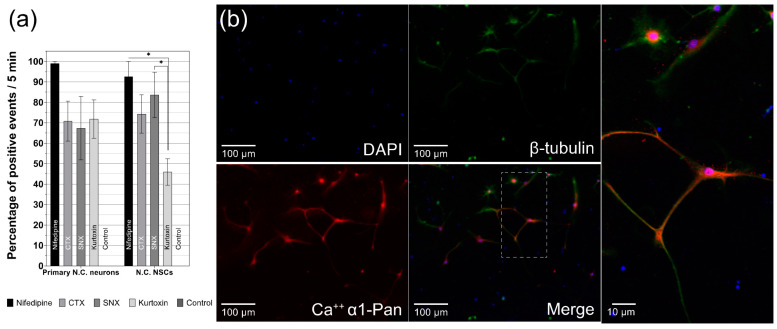
Analysis of the influence on spontaneous CN NSC calcium oscillations by different specific calcium channel modulators. (**a**) Percentage of the positive impact on spontaneous oscillations after applying the indicated substances over a measurement period of 5 min. (**b**) Representative photomicrograph of differentiated CN NSC: DAPI stains the cell nuclei, β-tubulin the cytoskeleton, immunocytochemical identification of the alpha-1C subunit of voltage-dependent L-type calcium channels. Data are shown as mean ± SEM; asterisks indicate the significance level: * *p* < 0.05.

The analysis of the Ca^2+^ channel reactivity of differentiating CN NSCs versus primary CN Neurons was compared with their CaI results (Figure 6a). The cells examined were then fixed for the immunocytochemical analysis (Figure 6b). It was shown that the L-type Ca^2+^ channel inhibitor nifedipine had a strong positive effect on the spontaneous Ca^2+^ oscillations in primary neurons in 99 ± 2.5% (*n* = 44) of the analyzed cells—comparable to CN NSC with a mean of 92.50 ± 7.4% (*n* = 14) positive influence (mean ± SEM). The P-, Q-, and N-type calcium channel blocker conotoxin (CTX) caused 70.8 ± 9.8% (*n* = 49) of primary neurons and 74.3 ± 9.4% (*n*= 40) of CN NSCs result in an increased rate of Ca^2+^ oscillations. The R-type calcium channel inhibitor SNX-482 significantly increased the spontaneous excitation rate in 67.4 ± 15.5% (*n* = 38) of the cases in neurons and 83.7 ± 11.1% (*n*= 35) of the cases in NSCs. With the T-type calcium channel blocker kurtoxin, the rate of increase in the CaI measurement of neurons was 71.83 ± 9.4% (*n* = 63), and of CN NSCs at 46 ± 6.5% (*n* = 26). In the control groups, both primary CN Neurons and NSCs did not show any significant influence on the spontaneous Ca^2+^ excitation rate (*n* = 53). Significant differences in the positive influence of Ca^2+^ channel inhibitors only were found in CN NSCs induced by nifedipine vs. kurtoxin and SNX vs. kurtoxin—with kurtoxin having the significantly lowest influence rate on the oscillation frequency. The most significant influence in both primary CN neurons and differentiated NSCs resulted from nifedipine (Figure 4a). In all differentiated NSCs analyzed, just like in primary neurons, voltage-dependent calcium channels (α-1-subunits) in the somata and cell processes were stained immunocytochemically (Figure 6b).

## 4. Discussion

The discovery of postnatal and adult neurogenesis [70] led to the investigation into ways to influence NSCs proliferation, migration, and differentiation [72,73,74]. Spontaneous activity already affects neurogenic processes in the very early stages. It can direct gene expression, proliferation, migration, axonal and dendritic growth, differentiation, and cell fate [75]. This spontaneous activity is caused by ion channels expressed at an early stage, which can be detected in neuronal precursor cells during differentiation, similar to embryonic neurogenesis [76,77]. Voltage-dependent calcium channels influence the determination of stem-cell fate and promote neurogenesis through the expression of NeuroD [58]. Spontaneous neuronal activity is essential for the developing auditory pathway in the pre-hearing period [78,79]. It offers possible approaches for the treatment of congenital or acquired hearing disorders. In the cochlear nucleus, the second neuron of the auditory pathway, a quiescent stem-cell potential was detected early postnatally and in the adult animal [24,26]. Cells isolated from this were able to develop progenitor cells and proliferate indefinitely. According to differentiation protocols, the cell differentiation into neuronal and glial cells was induced by the withdrawal of growth factors [69].

The present study aimed to analyze the calcium-triggered cell activity of neural stem cells from the rat cochlear nucleus. The results indicate that the differentiation phase is characterized by a significant increase in spontaneous calcium oscillations in individual cells and their compartments (Figure 1 and Figure 2). Spontaneous oscillations initially occurred primarily within the somata, while after a phase of differentiation, axons and dendrites show the highest rate of spontaneous discharges. The analysis of the growth cones also shows this pattern. Spontaneous bursts were affected by different types of voltage-dependent calcium channels (VDCC). The isolated CN neural stem cells showed a pattern comparable to primary neurons in the brainstem nucleus (Figure 5).

### 4.1. CN NSC Differentiation Assay and Calcium Imaging Allow In Vitro Analyses in the Longitudinal Course

Since there are no specific markers for neural stem cells, general characteristics were defined, which were valid across species and regions [80,81]. Neural stem cells have three cardinal properties: (a) They can renew themselves indefinitely through mitosis and (b) can produce daughter cells (progenitor cells) independently of themselves. In addition, (c) they have the property of multipotency, i.e., they develop into all cell forms of the neuroectodermal line (neurons, astrocytes, and oligodendrocytes). Progenitor cells of a stem cell, in turn, can assume a stem-cell function or differentiate. However, they only have a limited ability to renew themselves and can develop at least two different cell lines. The cell cultures obtained were examined in detail in previous studies based on these defined characteristics to analyze the cochlear nucleus [24,26,82]. In cell culture, the cells used had the property of unlimited cell division and self-renewal. After the withdrawal of the growth factors used, the progenitor cell markers nestin [82,83], doublecortin [84,85], Sox-2 [86,87,88], and Atoh-1 [89] were detected. Neuronal and glial cell markers were only detectable after a differentiation phase.

For the culture and propagation of CN quiescent neural stem cells, a cell culture system was used, which is specially adapted to neural stem and progenitor cells. To control the influencing factors, the cultivation was carried out in a serum-free medium (Neurobasal, B-27 supplement, and GlutaMAX) with the mitogens EGF and FGF-2. These diffusible factors are crucial for the induction, mitotic reproduction, and survival of NSCs and progenitor cells [81,90,91,92]. The mitogens and the conditioning of the cell cultures with a continuous supply of the factors play a unique role [93]. As in embryonic development, these are essential for adult neurogenesis [94]. A neurosphere assay was used, which is a well-established method to analyze quiescent stem-cell potential in vivo in neuronal tissue [27,80,95,96]. The previously described DIF medium has been used successfully on stem cells, and differentiation protocols from the precedent studies on CN cell maturation were induced by withdrawing growth factors and adding retinoic acid (all-trans retinoic acid, Tretinoin) (Figure 1b). Retinoids bind to specific receptors in undifferentiated cells in the cell nucleus and can influence maturation [97]. These processes are already well-established in vitro.

The calcium imaging technique allows the intracellular calcium flow to be visualized with the aid of indicator dyes [98]. These dyes are chelating molecules that bind calcium selectively and emit a signal via a fluorescent side. With high-speed photodetectors, intracellular calcium dynamics can be detected and quantified very sensitively. This live imaging method enables analyses that can also display intracellular concentration gradients. Another advantage is the ability to scan multiple cells simultaneously (Figure 4d) and thus examine influences in the screening process or even cell interaction in the network. The calcium imaging system was adapted to CN NSCs to achieve an optimal signal-to-noise ratio for the oscillation frequencies. Therefore, BAPTA-1 (Oregon Green, O.G.) was used, proving itself in vitro in a similar constellation [98]. In preliminary experiments, different calcium-sensitive, membrane-permeable fluorophores were systematically tested on single-cell cultures of CN NSCs. Fura-2, AM (F1221, Thermo Fisher Scientific) was the focus because it enables ratiometric investigations to reduce artifacts from bleaching, focus drifts, background activity, and the excitation fluorescence intensity. No stable results were achieved with this dye in CN NSCs or the primary CN cultures. Therefore, alternatives were investigated which have different binding affinities for calcium in vitro due to different dissociation constants: K_d Fura-2/AM_: 145 nM, K_d Fluo-4 FF_: 345 nM, K_d Oregon Green BAPTA-1_: 170 nM [99]. It was found that the calcium-binding affinity of Fura-2/AM in CN NSCs is not ideally suited to map the specific frequency of the oscillations of these cells. Fluo4FF, AM (F23981, Thermo Fisher Scientific), whose calcium-binding affinity is significantly lower, did not deliver reproducible results with CN NSCs either. However, Oregon Green 488 BAPTA-1 (O6806, Thermo Fisher Scientific) showed an optimal Ca-binding affinity for CN NSCs.

The calcium indicators Fura-2 and Oregon Green 488 BAPTA-1 on the one hand differ slightly in their dissociation constant (K_d_). They also have different values of their calcium-binding rate (K_on_), their calcium dissociation rate (K_off_), and equilibrium times (τ) [3,4]. This leads to different rise and decay times of the fluorochromes concerning their calcium-binding rate, different strengths and speeds of calcium-binding, and thus slightly different optimal oscillation frequencies. This could explain why certain calcium-sensitive fluorochromes are better suited to visualize calcium activity in specific cell types in particular stages of activity. Reproducible and reliable measurements were thus possible with this dye on the cell cultures described. Oregon Green offers a high signal-to-noise ratio and is, therefore, one of the most frequently used dyes for the qualitative determination of intracellular calcium concentration [100].

### 4.2. CN NSC Differentiation Is Associated with Increased Spontaneous Activity and Specific Intracellular Patterns

For the differentiation period from DIF d0 to d4, there was a significant increase in spontaneous calcium oscillations from an average of 1.9 ± 2 to 23.4 ± 0.5 within the measurement period of 300 s, and thus an increase of approximately 1232% (Figure 1a). Calcium homeostasis and the exchange of ions between intra- and extra-cellular space are relevant for neuronal cell differentiation [101]. The maturation of neural stem and progenitor cells in vitro and in vivo is characterized and dependent on an increase in spontaneous neuronal activity [102]. In addition to the structural formation of neuronal regeneration and neurogenesis, cell differentiation also depends on these spontaneous activities. They are, therefore, an essential part of neural development. In vivo analyses show that in the increased spontaneous activity of neural stem cells, a halt of cell migration and an impulse for dendrite formation is induced [102]. Analyses of CN NSCs showed that at the induction time of the differentiation at DIF d0 in the somata, the highest spontaneous activity was present, with approximately 2.4 ± 0.3 excitations per 300 s, while they were significantly lower in axonal and dendritic extensions. This ratio changed over the course of the differentiation time to DIF d4 in favor of the axonal branches. These showed 28.89 ± 1.4 excitations/300 s, while in the somata, 24.34 ± 0.56/300 s were determined. The dendritic cell processes also tended to show slightly higher excitation values of 27.47 ± 0.9/300 s (Figure 2a,b). Such a pattern has already been demonstrated in other regions in which neurogenesis occurs [103,104]. In early neuronal cell development, the spontaneous calcium activity is primarily dependent on gap junctions and voltage-dependent calcium channels; later activity becomes primarily dependent on neurotransmitters and synaptic formation. Therefore, the initiation and regulation of calcium activity in the early maturation phase is of great significance, especially for developing synapses. In later stages, the ion channels are involved in the regulation of neurotransmitter signals. Thus, the individual activity of calcium represents a possible relevant drive for maturation in the cell processes [46,105,106]. Conversely, the results show that a specific pattern of spontaneous calcium activity in CN NSCs is a sign of functional maturity [46]. Thus, the phenotypic maturation was achieved, and signs of neural activity were displayed, which further indicates the potential integration into existing neural networks, and therefore a possible future therapeutic approach. The relationship between spontaneous calcium oscillations for neuronal development, maturation, and cell migration has already been shown in the developing neocortex. Cell migration is dependent on the expression of voltage-controlled calcium channels and neuronal activity. If the rate of spontaneous excitations increases, cell migration in vivo is suppressed, and premature neuronal maturation and the formation of dendrites and synapses are initiated [107]. Bando et al. described an increase in neuronal calcium activity from PND0 to PND3. With PND0 frequencies averaging eight excitations per 10 min could be evaluated. In CN NSCs, comparable values were found at DIF d0. However, Bando et al. analyzed vibratome sections of mice, while in the present study on the CN, single cells obtained from rat NSC cultures were scanned.

The subventricular zone (SVZ) is the area with the main stem-cell niche of the adult mammalian brain [107]. SOC calcium channels were identified as particularly important for the SVZ NSC maturation processes. The pharmacological blockade of the channels led to a reduced proliferative potency in vitro [108]. If the SOC channels are blocked in vivo, this leads to a significantly reduced neurosphere formation in vitro. The modulation of the calcium oscillations did not influence the differentiation into the cell forms of the neuroectodermal line; however, NSC activation and self-renewal are suppressed by pharmacologically reduced spontaneous calcium excitations. In this in vivo and in vitro analysis of the mouse, calcium transients and the responsible channels were shown to play a decisive role for NSCs and may offer approaches for exogenously controllable neuronal repair mechanisms.

During differentiation of the somata and growth cones, significant differences were shown. In the analyses carried out separately, the somata showed an average of 18.9 ± 1.8 oscillations/300 s. The growth cones displayed 23.6 ± 1.02 spontaneous calcium oscillations/300 s (Figure 3a). Calcium signals significantly affect the motility and routing of growth cones during neuronal maturation [104]. Calcium regulation contributes considerably to the growth rate and the maximum outgrowth of axons. The axonal growth and thus the integration into existing neural structures is essentially dependent on the activity and calcium signals [109]. It showed that CN NSCs are potentially able to integrate functionally after induction—the essential prerequisite for causal therapy in the case of structural damage in the auditory pathway. Tang et al. described a dependence of the frequency and amplitudes of spontaneous calcium transients in growth cones on axonal outgrowth. In the in vitro analysis of developing cortical neurons, calcium oscillation frequencies between 0 and 2.5/min were measurable [110]. The values are comparable to the frequencies of CN NSCs—albeit a little lower. On the one hand, the differences could be explained by the different animal models—on the other hand by the diverse origins of the neurons. In addition, primary developing neurons were examined here instead of the propagated NSCs.

A series of 2-D in vitro cultures was carried out to analyze the growth cones, which were particularly suitable for identifying and displaying them. For this reason, a comparatively lower cell density was plated on the dishes (70 cells/mm^2^) for the investigation of GCs. Thus, in the analysis of growth cones within the differentiation time, d0-d4, slightly less-dense cell populations, and fewer cell–cell contacts formed. This factor may have a certain influence on spontaneous calcium oscillations since it is known that higher rates occur in neural networks than in isolated cells [111]. For this reason, oscillation rates were only compared statistically with one another in absolute values in those cases when the culture conditions were precisely the same. This factor would explain why different absolute values were measurable in the analysis of somata vs. growth cones (Figure 3) than in somata vs. axons vs. dendrites (Figure 2).

### 4.3. Voltage-Dependent Calcium Channels Cause Spontaneous Oscillations in the Early Phase of CN NSC Maturation and Allow Exogenous Influence

After showing signs of functional maturity from CN NSCs and their spatiotemporal changes were revealed, the question arose as to which channels are responsible and whether an exogenous influence is possible. For indirect analysis, specific calcium channel inhibitors were therefore added after the initiation of the CaI. The modulation of the L-type calcium channels by nifedipine and N-type calcium channels by ω-conotoxin (CTX) significantly affected spontaneous calcium oscillations (Figure 5). If these channels are blocked, the calcium outflow is reduced, and thus spontaneous excitations were increased. This effect was also demonstrated when examining primary differentiated neurons of the CN—and therefore gives indications that the inductions of the isolated neuronal stem cells differentiate into comparable functional cells as they are present in vivo. The R-type calcium channel blocker, SNX-482, and the T-type inhibitor, kurtoxin, showed comparable but somewhat lesser effects in CN NSCs. These also lead to significantly increased calcium oscillations and are therefore considered to have a functional influence on the maturing cell. This channel functionality also was shown in the primary neurons. The potential positive influence of the channel blockers on the oscillations of the cells is shown in Figure 6a. Here, positive events after the application of the channel blockers were analyzed in relation to the resting discharge frequency. In immunocytochemical analysis, voltage-dependent calcium channels were also detected after the differentiation phase of the CN NSCs (Figure 6b).

Calcium channel modulators are already being used therapeutically on a regular basis—e.g., representatives of dihydropyridines for regulating blood pressure [112] and, due to their excellent cerebrospinal fluid penetration, as antidementia drugs [113]. This therapy already affects the central nervous system, whereby in addition to influencing vascular dementia, neuroregenerative and neuroprotective effects also appear to be in action [113]. A functional effect through the modulation of neural activity has already been shown in stroke models [114]. For the treatment of epilepsy, modulators of the T-type, P- and Q-type calcium channels are in clinical use—such as ethosuximide, pregabalin, and gabapentin. An influence on the hippocampal neurogenesis of these substances has already been shown in animal models. It showed that ethosuximide induced NSC proliferation and positively influenced cognitive deficits in Alzheimer’s dementia. Additionally, in vitro influences on NSC differentiation were demonstrated [115].

In summary, CN NSCs show a pattern of spontaneous calcium oscillations, which allows conclusions to be drawn about axonal growth and cell migration. In addition, responsible voltage-dependent calcium channels were identified, which were also present in the same constellation in neurons, primarily derived from CN. The spontaneous activity can be modulated exogenously. Thus, the processes of neurogenic induction, migration, and maturation could be influenced. Results from the clinical use of calcium channel modulators and animal experiments give hope that a targeted therapeutic influence of these processes through exogenous substances may be possible.

### 4.4. Limitations of the Study

There may be some limitations within the present study. Spontaneous calcium oscillations play a role in neurogenesis, especially within developing neuronal networks. In the current study, primarily isolated, maturing cells were analyzed. These findings provide the basis for further, more complex investigations within networks. Individual cell compartments can best be assessed in individual cell assays. Another point is that the CN NSCs have not yet been analyzed electrophysiologically. The patch-clamp technique offers additional knowledge here—beyond changes in calcium concentration in the CaI. However, the advantages of CaI are that intracellular gradients can also be analyzed and that the cells are less subject to exogenous manipulation, so that their spontaneous oscillations can be observed more closely. The current data concentrate on early postnatal animals and must also be considered in the later stages of their age. The advantage of early postnatal cells is the particularly high stem-cell potential, so that sufficient propagation for systematic analyses is possible. Therefore, such cells were primarily examined to form the basis for further findings. In the present study, the primary focus was on the voltage-dependent calcium channels. Further ion channels and signal cascades may be particularly important in the further course of neuronal maturation. The initially active channels provide the basis for further analyses. In the present study, functional investigations on the voltage-dependent channels were only carried out on differentiated cells on DIF d4. At this in vitro point in time, the highly active cells appeared to be the most suitable for the functional investigations and investigations on the effects of different channel blockers—although analyses over time could provide further new findings.

## 5. Conclusions

The study results show that the neuronal differentiation of CN NSCs is accompanied by intracellular, spontaneous calcium activity. Early differentiation begins with a predominant calcium activity in the somata area. This characteristic changes over time and the axons and dendrites are most active around the point in time at which the neuron-specific intermediate filament β-III-tubulin is expressed. The same applies to the neural growth cones, which have significantly increased spontaneous excitations. These spontaneous oscillations are dependent on different voltage-dependent calcium channels, which can be exogenously influenced with the help of already-established selective calcium channel inhibitors. In comparison with differentiated primary CN Neurons, CN NSCs show a similar pattern. The silent CN stem-cell niche shows functional maturity through calcium oscillations in cellular maturation, which presumably influence neurogenesis, migration, and differentiation in a similar way to other areas. These findings may offer a basis for potential regenerative approaches in the future, to influence the behavior and function of neural stem cells. A targeted interference of the cellular maturation processes might provide the basis for a therapeutic approach following damages within the auditory pathway structures.

## Data Availability

All data from the study are given in the manuscript.
